# Socioeconomic Inequalities in Psychological Distress among Urban Adults: The Moderating Role of Neighborhood Social Cohesion

**DOI:** 10.1371/journal.pone.0157119

**Published:** 2016-06-09

**Authors:** Özcan Erdem, Frank J. Van Lenthe, Rick G. Prins, Toon A. J. J. Voorham, Alex Burdorf

**Affiliations:** 1 Erasmus Medical Center, Department of Public Health, Rotterdam, The Netherlands; 2 Municipality of Rotterdam, Department Research and Business Intelligence, Rotterdam, The Netherlands; 3 Municipality of Rotterdam, Department Health Authority, Rotterdam, The Netherlands; 4 University of Applied Sciences, Department of Primary Health Care, Rotterdam, The Netherlands; University of Kwazulu-Natal, SOUTH AFRICA

## Abstract

**Background:**

Various studies have reported socioeconomic inequalities in mental health among urban residents. This study aimed at investigating whether neighborhood social cohesion influences the associations between socio-economic factors and psychological distress.

**Methods:**

Cross-sectional questionnaire study on a random sample of 18,173 residents aged 16 years and older from 211 neighborhoods in the four largest cities in the Netherlands. Psychological distress was the dependent variable (scale range 10–50). Neighborhood social cohesion was measured by five statements and aggregated to the neighborhood level using ecometrics methodology. Multilevel linear regression analyses were used to investigate cross-level interactions, adjusted for neighborhood deprivation, between individual characteristics and social cohesion with psychological distress.

**Results:**

The mean level of psychological distress among urban residents was 17.2. Recipients of disability, social assistance or unemployment benefits reported higher psychological distress (β = 5.6, 95%CI 5.2 to 5.9) than those in paid employment. Persons with some or great financial difficulties reported higher psychological distress (β = 3.4, 95%CI 3.2 to 3.6) than those with little or no financial problems. Socio-demographic factors were also associated with psychological distress, albeit with much lower influence. Living in a neighborhood with high social cohesion instead of low social cohesion was associated with a lower psychological distress of 22% among recipients of disability, social assistance or unemployment benefits and of 13% among citizens with financial difficulties.

**Conclusions:**

Residing in socially cohesive neighborhoods may reduce the influence of lack of paid employment and financial difficulties on psychological distress among urban adults. Urban policies aimed at improving neighborhood social cohesion may contribute to decreasing socio-economic inequalities in mental health.

## Introduction

Depression is a common health problem among adults in European countries, including the Netherlands [1, 2]. Depression almost doubles the risk of premature mortality [3], and seriously affects quality of life of patients [4] and of those in their immediate environment [5]. It was the fourth leading contributor to the global burden of disease in Europe in 2010 [6], and is expected to be the leading contributor in 2030 [7]. Preventing the onset is an important strategy to reduce the public health burden of depression [8].

The risk of depression is unequally distributed across the population. Those with a low income [9], low level of education [9, 10] or without a job [11] are at an increased risk of depression. The largest cities in the Netherlands host relatively many residents from lower socioeconomic groups [12], which may be one of the reasons of a higher prevalence of depression in those cities (12–15%) as compared to the overall prevalence in the Netherlands (10%) [13]. Explanations for these inequalities include poor material circumstances, lack of social support, and unhealthy behaviors [14]. However, these individual factors cannot entirely explain the observed between-neighborhood variation in depression in the Netherlands. This suggests that neighborhood differences in depression are not exclusively attributable to compositional effects, i.e. clustering of residents with low socio-economic characteristics in poor neighborhoods, but may also be due to contextual effects of neighborhood features. A rapidly increasing literature points towards the role of social contextual determinants of depression [15, 16] and a role for such determinants in the explanation of inequalities in health outcomes in general. One of these factors is neighborhood social cohesion, which refers to the extent of connectedness and solidarity between and among neighbors in society. In socially cohesive neighborhoods the relationships between neighbors are strong and the willingness to help each other is present. Neighborhood social cohesion is a feature of the community which affects all members of the neighborhood, and distinctively different from individual-level social networks and social support [17].

Studies have shown mental health benefits of residing in a socially cohesive neighborhood [18–20]. Living in economically deprived neighborhoods with a strong social cohesion was associated with better mental health than living in economically deprived neighborhoods with low levels of social cohesion [21], suggesting a buffering role of neighborhood social cohesion.

Several studies on social cohesion, social capital and health have presented some evidence for health benefits of residing in a socially cohesive neighborhood. There are at least three plausible pathways by which social cohesion or social capital can affect individual health [17]. Firstly, social capital may affect health by both facilitating more rapid diffusion of health related information and promoting healthy norms of behavior (e.g. jointly walk with neighbors in a nearby park) or exerting informal social control over unhealthy behaviors (e.g. adolescent drinking, smoking, and drug abuse). Secondly, social capital may affect health by stimulating co-operation between residents to ensure access to local (health) services and amenities. These could include local pressure groups who are lobbying for the provision of services (e.g. playgrounds, recreational facilities, green area, community health clinics). Thirdly, psychosocial processes are a way in which social capital may affect health by giving emotional support (e.g. alleviate the pain of stressful life events) and enhancing self-esteem and mutual respect. Hence, higher levels of neighborhood social cohesion or social capital may buffer the impact of unfavorable material and social circumstances on depression among lower socioeconomic groups residing in urban areas.

Specifically, we hypothesize that (i) lower educated people, those with financial difficulties and lack of employment residing in the four largest cities of the Netherlands are at an increased risk of depression as compared to those with higher educational level, a higher income and with a job and (ii) that higher levels of neighborhood social cohesion buffer the relation between individual socio-economic factors and depression.

## Methods

### Study design

All municipalities in the Netherlands are required by law to gain insight into the health of the local population every four years. In 2008, the municipal health services of the four largest cities (Amsterdam, The Hague, Rotterdam and Utrecht) in the Netherlands jointly conducted a survey on physical and mental health, social well-being, lifestyle, health care use and demographics of their inhabitants. This survey was linked at neighborhood level to external surveys on social cohesion and on social deprivation.

### Sampling

In each city a random sample was drawn from the municipal population registers aged 16 years and older, stratified by district and age. A total of 42,686 respondents received an invitation. Although no formal power calculation was conducted, this sample size was considered sufficiently large to have at least 100 respondents per neighborhood. In the Netherlands each citizen is legally required to register his home address in a municipal register and municipal registers are collated to avoid multiple registration. Respondents were asked to fill in a written or web-based questionnaire or to take part in a personal interview when having difficulties to complete the questionnaire. Extra effort was made to target vulnerable groups, i.e. older Turks and Moroccans with limited language skills and residents of neighborhoods with a low response in previous surveys. Non-responders were contacted by telephone or visited at their home and were offered personal help to fill in the questionnaire in the language used by the respondent e.g. in Turkish or Arabic.

### Response

The overall response was 49% (n = 20,877); 54% in Utrecht, 51% in The Hague, 50% in Amsterdam and 47% in Rotterdam. Response was higher among women than among men and increased with age. The response was highest among Dutch (57%) and lowest among Moroccans (30%) [22]. We omitted 12.8% of the respondents as a consequence of at least one missing item on socio-demographic characteristics, neighborhood cohesion and deprivation, and psychological distress. The final sample for analysis was 18,173 respondents. These respondents lived in one of 211 neighborhoods (on average 86 respondents (SD: 63) per neighborhood).

### Definition of a neighborhood

For social cohesion it is important that the definition of a neighborhood is a functional social entity. Previous research has found that there is a sense of community in Dutch neighborhoods which were defined by a 4 digit postcode [23]. These neighborhoods are often named (e.g. "Delfshaven" or "Vreewijk") to which people identify themselves. Therefore, we defined neighborhoods based on the 4 digit postcode. In the Netherlands, there are about 4,000 neighborhoods. These areas comprise on average of approximately 4,000 residents.

Ethical approval was not required as this study relied on secondary anonymized data collected in the context of performing statutory tasks (Public Health Act of the Netherlands), in strict accordance with the national standard [24]. Respondents were informed by letter that by filling out the questionnaire they gave permission for use of anonymous data for research aimed at improving population health in their place of residence. Respondents were contacted through municipal health services and in the dataset available for research purposes all identifying information has been removed. All research activities adhered to the regulations of the Dutch Code of Conduct for Medical Research.

### Measures

#### Psychological distress

This study used psychological distress as an indicator of depression [25, 26], measured with the Kessler Psychological Distress Scale (K10). The K10 has been developed as a screening instrument for psychological distress in the general population [27]. The K10 discriminates DSM-IV disorders from non-cases [26] and is strongly associated with the Composite International Diagnostic Interview (CIDI) diagnosis of anxiety and affective disorders [25]. In a recent Dutch study, the K10 proved to be reliable (Cronbach’s: 0.94) and valid (area under the curve (*AUC*: 0.87)) in detecting any depressive disorders. At the cut-off of 20 points, sensitivity (0.80) and specificity (0.81) are sufficiently high to appreciate the K10 as appropriate screening instrument [28].

The K10 scale consists of 10 questions that measure a person’s level of anxiety and depressive symptoms in the previous four weeks. The items included were: “Did you feel …1) tired out for no good reasons?”, 2) nervous?”, 3) so nervous that nothing could calm you down?”, 4) hopeless?”, 5) restless or fidgety?”, 6) so restless that you could not sit still?”, 7) depressed?”, 8) that everything was an effort?”, 9) so sad that nothing could cheer you up?” and 10) worthless?”. Each item has five response categories "none of the time", "a little of the time", "some of the time", "most of the time" and "all of the time". Cronbach’s alpha was 0.92, therefore a sum-score was calculated (range 10–50), with higher scores reflecting more psychological distress.

#### Socio-demographic and socioeconomic factors

Gender, age, ethnicity, marital status and years of residence in their current city were derived from the questionnaires. Ethnicity was defined based on country of birth of parents and the respondent, according to the standard definition of Statistics Netherlands [29]. Marital status was categorized into widow or widower, divorced, unmarried or never been married and married or living together. To control for duration of exposure to neighborhood context, years of residence in their current city was included in the analysis, measured by the question "Since what year do you live in your current city?" For the analysis, we constructed four categories (0–5 years, 6–15 years, 16–25 and 26 or more years).

Socioeconomic position was measured by the highest educational level attained, categorized into primary school, lower general secondary education, higher general secondary education and college or university. Employment status distinguishes the categories student, housewife or houseman, recipient of social benefits (disability, social assistance, unemployment), (early) pensioner and (self-)employed. Whether people experienced financial deprivation was measured by the question "Have you had difficulty in the past year to make ends meet with the household income?" with answers on a 4-point scale ranging from “great difficulty” to “no difficulty”. It was categorized into two levels, by taking “great” and “some”, and “almost no” and “no” together.

#### Neighborhood social cohesion

The dataset on neighborhood social cohesion was obtained from WoON 2009, a national survey among ~ 78,000 (response = 59%) randomly selected inhabitants of 18 years and older in the Netherlands (Ministry of Housing, Spatial Planning and Environment) [30]. At the individual-level, it was measured by five questions: “the people in my neighborhood get along well with each other”, “I live in a close-knit neighborhood with a lot of solidarity”, “I have a lot of contact with my direct neighbors”, “I have a lot of contact with other neighbors”, “In this neighborhood, the people hardly know each other”. All questions were answered on a 5-point scale ranging from”totally disagree” to “totally agree”. The codes of the latter question were reverse-coded. This scale was interpreted a continuous variable with a higher score reflecting a higher social cohesion.

We conceptualized social cohesion to be a neighborhood construct. Commonly such measures are aggregated to a neighborhood level by taking the mean of the items measured at the individuals living in the neighborhoods. However, one of the major disadvantages of this method is that these variables are subject to individual perception. This perception is likely to be influenced by characteristics of the individual (e.g. gender, ethnicity, age). Another disadvantage is that by taking a simple mean value, the reliability of the aggregated measure differs between neighborhoods, because it is likely that there are more respondents in one neighborhood than in the other. Finally, the separate items that measure social cohesion are not independent of each other, but nested within individuals. Therefore, the response to one item is likely to be strongly associated with a response on another item. These disadvantages are circumvented by using the ecometrics method, as described by Raudenbush and Sampson [31–34]. This method accounts for the nesting of social cohesion items within individuals, who in turn are nested within the neighborhoods. A three-level linear regression model was used in which the item scores were the outcome. So, each response to each question by each participant was a separate row in the dataset. A categorical variable, indicating these five social cohesion items was included as level 1 predictor (items nested within individuals and neighborhoods). The model was adjusted for six level 2 predictors (individual variables nested within neighborhoods) that may influence the perception of social cohesion: gender, ethnicity, age, education, type of housing the adolescent lives in and years living in the current home. The residuals at the neighborhood level, which represent the deviations of the outcome scores at the neighborhood level from the overall mean value at the neighborhood level (and on which the neighborhood variance in the model is based), form the part that cannot be attributed to individual response patterns. In other words, these values represent the social cohesion variable at the neighborhood level that cannot be explained by individual response patterns. Positive values indicate higher than average levels of social cohesion.

Ecometrics also allows to assess the reliability of the social cohesion measure, using the variance at all levels (variance between neighborhoods, between individuals and between items) [33]. In our study, the reliability, which has a similar interpretation as Cronbach’s α was 0.66, which is considered acceptable [35]. For the analysis the neighborhood social cohesion measure was dichotomized at the mean into high and low social cohesion.

Sensitivity analysis showed almost the same scores on mean psychological distress when another classification of neighborhood social cohesion (the 33rd percentile versus the 66th percentile) was used.

#### Neighborhood confounder

Neighborhood deprivation was treated as a confounder since it was associated with psychological distress and neighborhood social cohesion [36]. Hence, we controlled for neighborhood deprivation to estimate the specific contribution of neighborhood social cohesion effect to psychological distress. The scores on neighborhood deprivation (2010) was obtained from The Netherlands Institute for Social Research (SCP), and were based on the average level of income, employment rate, and average level of education in each four digit postal code [37]. A higher score reflects a higher social disadvantage.

### Data analysis

Descriptive statistics were used to show the distribution of individual-level factors and to describe the neighborhood-level factors in the study sample (Table 1). Descriptive statistics of the outcome measure, psychological distress, and the percentage distribution of the variables in the sample were calculated using SPSS Complex Samples, weighting for gender, age and city district. Subsequently, to simultaneously examine individual-level and neighborhood-level predictors with psychological distress, multilevel linear regression analysis was fitted [38]. We started with an intercepts-only model to test for significant variance in psychological distress between the neighborhoods. Eight individual-level factors were added to test whether the variance could be accounted for by socio-demographic and socioeconomic factors. Neighborhood deprivation and neighborhood social cohesion were included in the model to test the association between neighborhood social cohesion and psychological distress adjusted for neighborhood deprivation. The associations of individual-level and neighborhood level factors with psychological distress are presented in Table 1. All interactions between neighborhood social cohesion and socio-demographic and socioeconomic factors in relation to psychological distress were tested. In case of significant interactions, combined factors were used in further analysis.

**Table 1:** Sample characteristics of 18,173 adults residing in neighborhoods (n = 211) in the four largest cities in the Netherlands in 2008 and their associations^b^ with psychological distress.

***Neighborhood level***			**Mean**	**SD**	**Min.**	**Max.**
Neighborhood deprivation			0.46	1.69	-2.95	5.24
Neighborhood social cohesion			-0.20	0.19	-0.70	0.24
***Individual level***			**Mean**	**SD**	**Min.**	**Max.**
Psychological distress	Weighted^a^		17.16	6.97	10	50
Psychological distress	Unweighted		17.15	6.99	10	50
		**Weighted**^a^	**Un-weighted**			
**Multilevel regression results**^f^		**percent**	**percent**	**β**^c^	**(95%CI)**	
**Socioeconomic factors**						
Education	Primary school	11.8%	15.0%	**1.56**	(1.22 to 1.89)	
	Lower general secondary education	25.3%	28.7%	**0.39**	(0.13 to 0.66)	
	Higher general secondary education	25.7%	24.2%	0.14	(-0.11 to 0.40)	
	College, university	37.1%	32.1%	ref.		
Employment status	Student	9.1%	9.0%	-0.16	(-0.55 to 0.23)	
	Housewife, houseman	6.7%	8.5%	**0.66**	(0.26 to 1.07)	
	Recipients of benefits^d^	10.1%	10.2%	**5.59**	(5.24 to 5.94)	
	(Early) retired	12.9%	19.0%	**0.95**	(0.53 to 1.37)	
	(Self-)employed	61.3%	53.3%	ref.		
Financial deprivation	Great, some financial difficulty	28.1%	26.3%	**3.38**	(3.16 to 3.61)	
	(Almost) no financial difficulty	71.9%	73.7%	ref.		
**Socio-demographic factors**						
Gender	Man	48.6%	43.8%	**-1.50**	(-1.69 to -1.30)	
	Woman	51.4%	56.2%	ref.		
Age	16–34 years	34.8%	30.7%	**1.22**	(0.72 to 1.71)	
	35–54 years	37.4%	30.8%	**1.21**	(0.76 to 1.65)	
	55–64 years	13.7%	16.6%	**-0.43**	(-0.83 to -0.04)	
	≥ 65 years	14.1%	21.9%	ref.		
Ethnic background	First generation non-Western	18.2%	16.7%	**1.00**	(0.71 to 1.28)	
	Second generation non-Western	5.1%	4.5%	**0.92**	(0.43 to 1.40)	
	Western	11.5%	10.8%	**0.53**	(0.23 to 0.83)	
	Native Dutch	65.2%	68.0%	ref.		
Marital status	Widow, widower	4.8%	7.7%	**1.36**	(0.97 to 1.75)	
	Divorced	8.4%	8.8%	**1.25**	(0.91 to 1.60)	
	Unmarried, never been married	30.2%	26.8%	**0.72**	(0.47 to 0.97)	
	Married, living together	56.6%	56.6%	ref.		
Years of residence in place	0–5 years	17.3%	15.6%	ref.		
	6–15 years	21.0%	18.2%	0.15	(-0.17 to 0.48)	
	16–25 years	17.8%	16.2%	0.12	(-0.22 to 0.46)	
	≥ 26 years	43.9%	50.0%	**0.42**	(0.09 to 0.74)	
**Neighborhood factors**						
Neighborhood deprivation^e^				0.07	(-0.04 to 0.19)	
Neighborhood social cohesion^e^	Low cohesion			**0.26**	(0.04 to 0.49)	
	High cohesion			ref.		

CI = confidence interval.

^a^ Calculated using SPSS Complex Samples, weighted for gender, age and city district.

^b^ These results are based on multilevel regression analysis.

^c^ Bold values are significant (p<0.05). Betas represent difference in mean psychological distress as compared to the reference category.

^d^ Recipients of disability, social assistance or unemployment benefits.

^e^ Neighborhood deprivation is in z-score units (per 1 SD increase). Neighborhood social cohesion is dichotomized at the mean into high and low social cohesion.

^f^ Intraclass correlation (%): 0.15.

For each model, the intraclass correlation coefficient (ICC), representing the proportion of total variability in psychosocial distress that is attributable to the neighborhoods, was calculated. These analyses are presented in Table 2. All analyses were performed in SPSS 19. Results were considered to be statistically significant at p<0.05.

**Table 2:** Multilevel regression analysis of psychological distress by interactions between neighborhood social cohesion with financial deprivation (Model 1) and employment status (Model 2).

	Model 1 β^b^ (95%CI)			Model 2 β^b^ (95%CI)		
Constant	**14.05**	(13.50 to 14.61)		**14.03**	(13.47 to 14.59)	
**Neighborhood factor**						
Neighborhood deprivation^a^	0.08	(-0.04 to 0.19)		0.08	(-0.04 to 0.19)	
**Interactions with financial deprivation**						
low cohesion x financial deprivation	**3.70**	(3.38 to 4.02)				
high cohesion x financial deprivation	**3.22**	(2.90 to 3.54)				
low cohesion x no financial deprivation	0.18	(-0.07 to 0.43)				
high cohesion x no financial deprivation	ref.					
**Interactions with employment status**						
low cohesion x student				-0.15	(-0.67 to 0.37)	
high cohesion x student				0.06	(-0.46 to 0.58)	
low cohesion x housewife, houseman				**0.71**	(0.17 to 1.26)	
high cohesion x housewife, houseman				**0.84**	(0.32 to 1.37)	
low cohesion x recipients of benefits^c^				**6.25**	(5.79 to 6.70)	
high cohesion x recipients of benefits^c^				**4.89**	(4.37 to 5.41)	
low cohesion x (early) retired				**1.19**	(0.68 to 1.69)	
high cohesion x (early) retired				**0.97**	(0.49 to 1.44)	
low cohesion x (self-)employed				0.21	(-0.07 to 0.50)	
high cohesion x (self-)employed				ref.		
**Random parameters**						
Variance neighborhood level (estimates and s.e.)	0.06 (0.05)			0.06 (0.05)		
Intraclass correlation (%)	0.14			0.14		

CI = confidence interval.

^a^ Neighborhood deprivation is in z-score units (per 1 SD increase).

^b^ Bold values are significant (p<0.05). Betas represent difference in mean psychological distress as compared to the reference category. In Model 1 is adjusted for gender, age, ethnicity, marital status, education, employment status and years of residence. In Model 2 is adjusted for gender, age, ethnicity, marital status, education, financial deprivation and years of residence.

^c^ Recipients of disability, social assistance or unemployment benefits.

## Results

### Study sample

The study sample consisted of relatively high percentage women (56%), native Dutch (68%), married or living together (57%), persons with college or university education (32%), employees or self-employed (53%), persons without or with almost no financial difficulties (74%) and persons residing for more than 26 years in their city (50%) (Table 1).

The intercepts-only model shows that 97.13% of the random variation occurred at the individual-level and 2.87% at the neighborhood-level (ICC = 2.87%). After adjusting for the differences between the socio-demographic and socioeconomic factors, the ICC decreased to 0.23%, suggesting that the differences in psychological distress between neighborhoods were almost entirely attributable to the composition of the neighborhoods.

### Socio-demographic and socioeconomic factors and psychological distress

The mean level of psychological distress among urban residents was 17.2 (SD = 7.0) (Table 1). Recipients of disability, social assistance or unemployment benefits reported higher psychological distress (β = 5.6, 95%CI 5.2 to 5.9) than those in paid employment. Persons with some or great financial difficulties reported higher psychological distress (β = 3.4, 95%CI 3.2 to 3.6) than those with little or no financial problems. Socio-demographic factors were also associated with psychological distress, albeit with much lower influence. Men reported lower psychological distress than women (β = -1.5, 95%CI -1.7 to -1.3). Compared to married couples or cohabitants, widowed persons reported higher psychological distress (β = 1.4, 95%CI 1.0 to 1.7). Finally, first generation non-Western inhabitants reported higher psychological distress than Dutch inhabitants (β = 1.0, 95%CI 0.7 to 1.3).

### Neighborhood social cohesion and psychological distress

Adjusted for socio-demographic and socioeconomic factors, and neighborhood deprivation, residing in a neighborhood with low social cohesion was significantly associated with higher psychological distress (β = 0.3, 95%CI 0.0 to 0.5) (Table 1).

### Interactions between neighborhood social cohesion and socioeconomic factors

The interaction between neighborhood social cohesion and financial deprivation was statistically significant (Table 2, Model 1). With persons without financial deprivation living in a neighborhood with high social cohesion as reference, persons with financial deprivation living in a neighborhood with low social cohesion were more at risk of psychological distress (β = 3.7, 95% CI 3.4 to 4.0) than persons with financial deprivation living in a neighborhood with high social cohesion (β = 3.2, 95% CI 2.9 to 3.5).These results suggest that living in a neighborhood with high social cohesion instead of low social cohesion was associated with a 13% (= (3.70–3.22)/3.70*100%) lower psychological distress among citizens with financial difficulties.

The interaction between neighborhood social cohesion and employment status was also statistically significant (Table 2, Model 2). Compared to workers in paid employment living in a neighborhood with high social cohesion, recipients of a benefit living in a neighborhood with low social cohesion were more at risk of psychological distress (β = 6.2, 95% CI 5.8 to 6.7) than recipients of a benefit living in a neighborhood with high social cohesion (β = 4.9, 95% CI 4.4 to 5.4). Thus, unemployed and disabled citizens in neighborhoods with high social cohesion reported a 22% (= (6.25–4.89/6.25)*100%) lower psychological distress than those in neighborhoods with low social cohesion.

The interactions between neighborhood social cohesion and other individual-level factors in relation to psychological distress were non-significant (not shown).

## Discussion

### Main results

This study showed inequalities in psychological distress by employment status and levels of financial deprivation, with increased risks for unemployed and disabled persons and for those who experienced financial difficulties. Neighborhood social cohesion modified the associations between financial deprivation or employment status and psychological distress. Living in a neighborhood with a high level of social cohesion was associated with lower psychological distress among unemployed and disabled citizens and among citizens with financial difficulties. Findings from the current study contribute to knowledge how neighborhood characteristics can moderate relationships between subgroups of the population and mental health. Whereas we observed almost no effect of neighborhood social cohesion on psychological distress in the total population, there were strong differential effects within the population. People are influenced by their living environment and our findings showed that high neighborhood social cohesion provided mental health benefits for the economically deprived groups by partly buffering the adverse effects of being poor and unemployed on mental health. Hence, future studies are encouraged to distinguish between overall influence of social cohesion on all citizens in a neighborhood and differential influence of social cohesion on specific subgroups.

This study indicates that unemployed or disabled citizens and those who experienced financial difficulties have higher psychological distress if they reside in *less* cohesive neighborhoods in the four largest cities of the Netherlands. In the introduction three different mechanisms for the impact of social cohesion on health were presented. Applied to our study, it is most likely that neighborhood social cohesion buffered poor mental health of unemployed and disabled citizens, and citizens with financial difficulties directly by reducing levels of socioeconomic-related stress as a result of the existence of affective support. High neighborhood social cohesion seems related to fewer daily stressors [39] and trust and mutual respect seem related to better health [40]. Further research is needed to empirically demonstrate a mediating role of support between neighborhood social cohesion and mental health. Further, higher levels of neighborhood social cohesion may protect mental health indirectly by promoting physical activity such as joint walk in a nearby park or in green surroundings, which is good for health [34, 41, 42].

High neighborhood social cohesion did not significantly modify the associations between gender, age, ethnic background, marital status, education and, years of residence and psychological distress. One potential explanation is that the importance of the neighborhood for those at higher risk of psychological distress for these factors is less than for those with a lower income, without a paid job or disabled. For example, younger adults were at an increased risk of psychological distress, but the potential benefits of neighborhood social cohesion are not used, as networks of friends outside the neighborhood may be more important for them. Similarly, divorced and single persons may need to work to get an income, and as a result spend only little time in their neighborhood. This would also explain that those without a paid job, and therefore potentially often in the neighborhood benefit more from social cohesion as compared to those with a paid job. Indeed, in the Netherlands 19% of young persons (15–25 years), 17% of the singles and 16% of people with only primary education have no contact with their neighbors, compared with 12% of the total population. This percentage is even higher among non-Western immigrants, where 20% of this group have no contact with their neighbors [43]. So, neighbors who have no contact with each other do not expect any kind of support from each other. As a consequence, no social or financial support will be available to reduce mental health problems.

### Comparison to other literature

Only few studies examined effect modification by neighborhood social cohesion of the association between individual- or area-level factors and mental health. Our results are in line with the results of Fone et al. [21], who suggested that living within income deprived areas with more social cohesion in the United Kingdom was associated with better mental health than living within income deprived areas with less social cohesion. Contrary to our findings, Stafford et al. found that people living in deprived households or deprived neighborhoods in England and Scotland were at an increased risk of common mental disorders in neighborhoods with high levels of attachment compared with neighborhoods with low levels of attachment [44]. The last result showed how the association between neighborhood social cohesion and mental health might vary across population groups and that high neighborhood social cohesion is not always beneficial for mental health or protect against mental health problems. On the other hand, Abada et al. could not demonstrate that living in racially mixed neighborhoods with high social cohesion was associated with less depressive symptoms among adolescents in Canada [45].

Few studies have explored whether social cohesion modifies the association between individual-level factors and health or lifestyle outcomes. Cramm et al. showed that high neighborhood social cohesion may act as buffer against the adverse effects of being poor on the well-being of older adults (70 years and older) in the Netherlands [46]. This result can be explained by the fact that the importance of the neighborhood for older adults is more than for young and adults residents. In the Netherlands merely 8–9% of 65 years and older residents have no contact with their neighbors compared with 12% of the total population [43]. This suggests that older adults spend more time in their neighborhood and are better able to make use of potential benefits of neighborhood social cohesion. Robinette et al. showed that high neighborhood cohesion buffers the effects of daily stressors, include discrimination, on negative affect i.e. well-being among persons aged 30–84 years in the United States [39], while a study among Asian Americans found that high neighborhood social cohesion was associated with lower odds of smoking among men, suggesting that social cohesion was protective against smoking under men [47]. Poortinga found that most of the indicators of bonding and bridging (i.e. social cohesion) and linking social capital were significantly positively related to self-rated health, but he found no support for the hypothesis that these three different aspects of social capital help buffer against the detrimental influences of neighborhood deprivation on self-rated health in England [40]. It is possible that we can get the same conclusions, if we examine different aspect of social cohesion in relation to mental health. Investigations in the future should that prove.

### Neighborhood social cohesion as a function of neighborhood change

When interpreting these results we must not forgot that neighborhood environments are not static but subject to change over time. Some physical changes are consequences of revitalization and restructuring through local government. This may lead to displacement of lower income households in a neighborhood by higher income households, known as the process of gentrification. In turn, gentrification can have positive and negative consequences such as deconcentration of poverty but also displacement of long-term inhabitants that can change social processes in a neighborhood [48, 49]. Hence, neighborhood social cohesion itself can be a function of neighborhood change. Studies have shown that residential mobility seems to weaken the neighborhood social cohesion [50, 51], which in turn may be associated with increased depressive symptoms [49]. This cross-sectional study cannot investigate these mechanisms.

### Strengths and limitations

The use of multilevel regression analysis in a large sample of four major cities in the Netherlands allowed us to unravel the interactions of social cohesion with socio-demographic and socioeconomic factors above and beyond other individual-level factors. In addition, neighborhood deprivation and social cohesion were derived from other sources than the questionnaire survey on individual factors and psychological distress, which prevents same-source bias. With regard to the construction of neighborhood social cohesion, an ecometrics approach was used to arrive at neighborhood level constructs from individual data [31, 33].

Some limitations need to be taken into account when interpreting the results. Our cross-sectional study prohibits causal inference. For example, unobserved confounders may have resulted in biased estimates of neighborhood deprivation and social cohesion. Noise pollution from traffic and neighbors or socially insecure and unsafe areas may have caused both a lower neighborhood welfare, less social cohesion and higher levels of psychological distress. Exclusion of such factors may have resulted in an overestimation of the associations between neighborhood deprivation or neighborhood social cohesion and psychological distress. Further, while we included several factors that may influence allocation to living in deprived or affluent neighborhoods, and to neighborhoods with low and high social cohesion, persons living in such different neighborhoods may differ in other respects, such as personality factors. Thus, we cannot entirely rule out an overestimation of the importance of neighborhood deprivation and social cohesion. Yet, adjustment for education, employment status, ethnicity, marital status and financial deprivation may have tackled this problem already to a substantial amount. Further, selective migration may be responsible for some of the associations found. Depressed persons may have less energy to move away from more deprived neighborhoods or from neighborhoods with low social cohesion, and persons with less distress may more often move to more affluent and cohesive neighborhoods. Previous research has shown, however, that health is a relatively marginal reason for moving to another address [52]. Another limitation is the dichotomization of neighborhood social cohesion at the midpoint into low cohesion and high cohesion, which has led to loss of variation. However, the current approach facilitates direct comparison of the modification effect with other categorical determinants.

### Interpretation and conclusions

Although the mental health of the inhabitants in the four largest cities in the Netherlands depends to a large extent on the socio-demographic and socioeconomic characteristics of individuals and for a small part of neighborhood characteristics, subsequent analyses gave evidence that neighborhoods with high social cohesion may partly buffer the adverse effects of low socioeconomic status on mental health. But, neighborhood social cohesion does not affect us all equally. Living in neighborhoods with high social cohesion may be more beneficial for persons in financial difficulties and for unemployed or disabled than other groups in relation to health. So, our study gives evidence that living in neighborhoods rich in social cohesion is potentially of importance in protecting mental health of some socioeconomic vulnerable individuals. This could have implications for reducing depression among adults in urban cities. Although in the Netherlands, all citizens are assured of subsistence as a result of long-standing socio-economic policy, the economic position of citizens with a low socioeconomic status still requires attention. Policymakers need to be aware of the existence of financial disadvantaged groups who are at higher risk of depression as a result of residing in low social cohesion neighborhoods. Hence, urban policies should focus on improving social cohesion in low cohesive neighborhoods and attention is needed for improving the economic position of financially disadvantaged groups in low cohesive neighborhoods.

### What is already known on this subject

- Research has shown that neighborhood social cohesion is an important characteristic of the social environment, but little research has examined its association with depression or psychological distress.
- Strong evidence for an inverse association between neighborhood social cohesion and depression or psychological distress is still lacking.
- Especially, whether social cohesion modifies the association between individual-level factors and depression or psychological distress is understudied.

### What this study adds

- Neighborhood social cohesion moderates the associations between financial deprivation or employment status and psychological distress.
- Living in a neighborhood with a high instead of low social cohesion was associated with a lower psychological distress of 22% among unemployed and disabled citizens and of 13% among citizens with financial difficulties.
- Neighborhood social cohesion does not affect all persons equally. It may partly buffer the adverse effects of low socioeconomic status on mental health.
